# Successful Laryngotomy for Laryngismus Paralyticus

**Published:** 1891-12

**Authors:** S. J. J. Harger


### SUCCESSFUL LARYNGOTOMY FOR LARYNGISMUS PARALYTICUS.

By S. J. J. Harger, V. M. D.

As the value of any operation can best be determined by compiling statistics from cases occurring in the experience of individual practitioners, I will take the opportunity of reporting the following case. The patient, a bay coach-horse, 16·3 hands high and about twelve years of age, was sent to the veterinary department of the University of Pennsylvania by Dr. Wm. H. Ridge. He had been a roarer for three months without any apparent cause, and was gradually becoming worse. Ordinary exercise for one square developed a loud laryngeal sound. Blisters and alterations were employed, but without effect. Feeling certain that there was a paralysis of the laryngeal muscles on the left side, from pressure on the corresponding recurrent nerve, laryngotomy was the only remedy. Instead of etherization, two ounces of choral hydrate were administrated, which answered the same purpose.

After making an incision into the larynx in front and on the median line in the usual manner, the tampoon canula was inserted and the left arytenoid cartilage carefully dissected out and dislocated from the cricoid. The mucous membrane was incised very close to the border of the arytenoid, in order to leave as much of the former as possible. The vocal cord was cut off close at its attachment to the latter and left in place. After the hæmorrhage was arrested, the laryngeal cavity was packed with oakum sprinkled with iodoform and the external incision closed with three sutures. The following day, the sutures and packing were removed, the laryngeal cavity washed out with carbolized water and the canula taken out. The breathing was slightly noisy, which disappeared in a few days. Daily washing out with the syringe and the application of iodoform constitued the after-treatment. The discharge was small and the wound healed kindly, without exhuberant granulations. Three weeks after the operation, the external incision had closed, and slight exercise developed no noise. At the end of six weeks, the animal was able to do his usual, severe work without any indication of roaring.

The diagnosis of muscular paralysis was verified after opening the larynx, by the almost complete relaxation of the left vocal cord during aspiration.

In this case, I did not suture together the edges of the mucous membrane, which, however, I shall do in all my future operations of this kind. The great barrier to success in these cases has been the formation of excessive granulation from the large area of muscular tissue exposed. This can be prevented by uniting the edges of the mucous membrane with two or three sutures of cat-gut. Neglecting this procedure leads most frequently to failure; heeding it has been the great element of success to Möller.

I read an article in the November number of the JOURNAL OF COMPARATIVE MEDICINE AND VETERINARY ARCHIVES referring to the difficulty of dissecting the mucous membrane from the arytenoid cartilage in Möller's operation. This operator simply incises this membrane very closely around the circumference ot the cartilage, and then draws the incised edges together with sutures.

